# An Adequately Robust Early TNF-α Response Is a Hallmark of Survival Following Trauma/Hemorrhage

**DOI:** 10.1371/journal.pone.0008406

**Published:** 2009-12-22

**Authors:** Rajaie Namas, Ali Ghuma, Andres Torres, Patricio Polanco, Hernando Gomez, Derek Barclay, Lisa Gordon, Sven Zenker, Hyung Kook Kim, Linda Hermus, Ruben Zamora, Matthew R. Rosengart, Gilles Clermont, Andrew Peitzman, Timothy R. Billiar, Juan Ochoa, Michael R. Pinsky, Juan Carlos Puyana, Yoram Vodovotz

**Affiliations:** 1 Department of Surgery, University of Pittsburgh, Pittsburgh, Pennsylvania, United States of America; 2 Department of Critical Care Medicine, University of Pittsburgh, Pittsburgh, Pennsylvania, United States of America; 3 Center for Inflammation and Regenerative Modeling, McGowan Institute for Regenerative Medicine, University of Pittsburgh, Pittsburgh, Pennsylvania, United States of America; University of California Merced, United States of America

## Abstract

**Background:**

Trauma/hemorrhagic shock (T/HS) results in cytokine-mediated acute inflammation that is generally considered detrimental.

**Methodology/Principal Findings:**

Paradoxically, plasma levels of the early inflammatory cytokine TNF-α (but not IL-6, IL-10, or NO_2_^-^/NO_3_^-^) were significantly elevated within 6 h post-admission in 19 human trauma survivors vs. 4 non-survivors. Moreover, plasma TNF-α was inversely correlated with Marshall Score, an index of organ dysfunction, both in the 23 patients taken together and in the survivor cohort. Accordingly, we hypothesized that if an early, robust pro-inflammatory response were to be a marker of an appropriate response to injury, then individuals exhibiting such a response would be predisposed to survive. We tested this hypothesis in swine subjected to various experimental paradigms of T/HS. Twenty-three anesthetized pigs were subjected to T/HS (12 HS-only and 11 HS + Thoracotomy; mean arterial pressure of 30 mmHg for 45–90 min) along with surgery-only controls. Plasma obtained at pre-surgery, baseline post-surgery, beginning of HS, and every 15 min thereafter until 75 min (in the HS only group) or 90 min (in the HS + Thoracotomy group) was assayed for TNF-α, IL-6, IL-10, and NO_2_^-^/NO_3_^-^. Mean post-surgery±HS TNF-α levels were significantly higher in the survivors vs. non-survivors, while non-survivors exhibited no measurable change in TNF-α levels over the same interval.

**Conclusions/Significance:**

Contrary to the current dogma, survival in the setting of severe, acute T/HS appears to be associated with an immediate increase in serum TNF-α. It is currently unclear if this response was the cause of this protection, a marker of survival, or both. This abstract won a Young Investigator Travel Award at the SHOCK 2008 meeting in Cologne, Germany.

## Introduction

Traumatic injury/Hemorrhagic shock (T/HS) is a major source of morbidity and mortality worldwide [1]. It is ranked the fifth leading cause of death in all age groups in the United States, where it remains the leading cause of death in individuals under the age of 44 years of age [2]. Regardless of the mechanism of injury, HS is a leading cause of death following trauma [1], [3], [4]. Trauma and hemorrhage trigger a complex cascade of events associated with alterations in hemodynamic, metabolic, and inflammatory/immune responses that are largely orchestrated by cytokines and chemokines, among a host of factors [5]–[7]. Over the past two decades, much has been learned about how cytokines modulate inflammation and associated processes [8], [9]. However, the exact role of each cytokine in the setting of trauma/hemorrhage is still unknown [7]. Some cytokines appear to promote inflammation in the context of traumatic injury, whereas other cytokines suppress the activity of these nominally pro-inflammatory cytokines [10], [11]. Nonetheless, the current paradigm equates elevated inflammation with adverse outcomes [12], [13].

This notion is increasingly being challenged, given the beneficial aspects of a properly regulated post-injury inflammation, including the orchestration of defenses against infection, as well as signaling for proper tissue healing [14]. Determining the exact role of the acute inflammatory response in the setting of T/HS is a highly complex endeavor, given the redundant and interconnected nature of inflammatory processes. The initial progression of the systemic inflammatory response can have different manifestations, depending on the specific characteristics of the injury [15]; individual genetics [16], wherein a gene polymorphism within the regulatory sequence affects the levels of cytokine production [10], [17]; and the robustness of the response to tissue damage and ischemia [18]. To date, the “classical”, nominally pro-inflammatory (or more properly, T_H_1) cytokines include tumor necrosis factor–α (TNF-α), interleukin (IL)-1β, IL-2, IL-6, IL-8 [19]–[21] and IL-18 [22]. On the other hand, nominally anti-inflammatory (T_H_2) cytokines such as IL-10 counteract the effects of T_H_1 cytokines in various contexts [23], including severe hemorrhagic shock [24]. Overproduction of either pro-inflammatory cytokines or anti-inflammatory cytokines may result in organ dysfunction [25], but it is unclear if these outcomes are a function of the duration, magnitude, or rate at which acute inflammation is triggered.

We sought to clarify the role of acute inflammation in human trauma victims. Paradoxically, both morbidity and mortality were inversely proportional to the degree of TNF-α production in human trauma victims. Swine subjected to a modified Wiggers paradigm of hemorrhagic shock recapitulated this basic finding. Thus, we suggest that an adequately robust TNF-α response following trauma may protect from death.

## Materials and Methods

### Human Trauma

All human sampling was done following approval by the University of Pittsburgh Institutional Review Board and informed consent was obtained from each patient or next of kin as per Institutional Review Board regulations. Blood samples from twenty-three (19 survivors: 12 males and 7 females, and 4 non-survivors: 3 males and 1 female) human trauma victims of motor vehicle accidents (17) or falls (6) were studied. The overall demographics of the patients were as follows: age: 47±4 yo; Injury Severity Score (ISS): 25±3; intensive care unit length of stay: 9±2; total length of stay: 13±2; number of days on a ventilator: 4±1. Plasma was sampled within the first 6 h following trauma.

### Animal Preparation

This study was approved by the University of Pittsburgh Institutional Animal Care and Use Committee and conforms to United States National Institutes of Health guidelines for the care and use of laboratory animals. Animals were acclimatized for at least five days in the Animal facility before being used in the experimental study. Twenty-three juvenile female Yorkshire/Durock pigs weighing approximately 25 to 35 kg (31.6±3.8 kg) were fasted overnight with access to water *ad libitum*. In the morning, they were sedated with an intramuscular injection of Ketamine/Xylazine/Telazol (1.0 ml/22.72 kg) and then were anesthetized with halothane inhalation to facilitate endotracheal intubation.

### Surgical Preparation

The experimental hemorrhage protocol was designed to stimulate a dynamically changing clinical situation reproduced by modifying a Wiggers model using progressive, discrete bleeding episodes based on the animal's physiologic response. During surgical preparation, anesthesia was maintained with isoflurane 1.0%–2.0% (1.0%–1.5% end tidal concentration) while the animals spontaneously ventilated oxygen/nitrogen (21%/79%) through a semi-closed loop system.

### Surgery Groups

Three pigs underwent surgical cannulation of the neck and groin (controls for Group A) and 4 pigs underwent surgical cannulation with an anterolateral thoracotomy (controls for Group B).

### Surgery/HS Groups

Pigs subjected to surgery and HS were divided into two groups, according to the severity of surgical preparation: Group A underwent neck and groin surgical cannulation whereas Group B underwent neck and groin cannulation and left anterolateral thoracotomy. Under aseptic conditions, the right external jugular vein and carotid artery were identified through a surgical incision in the neck and cannulated with a Swan-Ganz catheter (for blood sample collection and pulmonary pressure measurements), as well as, a Millar catheter (for blood pressure measurements), respectively. The right femoral vein and artery were isolated through a surgical incision at the groin and were cannulated using a bleeding catheter and a triple lumen catheter (blood pressure measurements). Group B was subjected to a left anterolateral thoracotomy after cannulation and prior to hemorrhage, in which a pericardial window was done and ultrasonic flow probes were placed on the pulmonary artery and aorta. Finally, a pressure sensing catheter in the left atrium was inserted. Physiological data were displayed on an eight-channel clinical monitor (Hewlett-Packard).

### Hemorrhage and Resuscitation Protocol in Swine

The following arterial pressure–driven experimental hemorrhage protocol was performed to exhaust the animals compensatory capacity with respect to hemorrhage, and thus approach the individual boundaries of irreversibility: A modified Wiggers model was used in the bleeding protocol, in which swine were bled to a predetermined hemodynamic endpoint. At the end of surgery, the pig was stabilized for 15 to 30 min, at which time the experiment was initiated (time 0). At time 0, controlled arterial hemorrhage via the femoral vein was initiated using a roller pump at a rate of 60 ml/min. The volume of shed blood was collected in a closed reservoir, and shed blood was collected in a reservoir and its volume determined from reservoir weight. Hemorrhage was sustained to maintain a mean arterial pressure (MAP) between 30 to 40 mmHg, with re-initialization of bleeding if the animal was capable of compensating its MAP. Resuscitation was initiated when the MAP fell below 30 mmHg for 10 min, below 20 mmHg for 10 seconds or when the pig was capable of maintaining a MAP between 30 and 40 mmHg for more than 90 min after the last bleed. Volume resuscitation was initiated using Hextend (Hospira Inc, Lake Forest, IL) at a rate of 60 ml/h. After resuscitation, swine were observed for 2 h and then sacrificed. Surviving animals were those that were alive up to 2 h post-resuscitation, whereas those that died prior to 2 h post-resuscitation were defined as non-survivors.

### Collection of Porcine Blood Samples

The first blood sample was a venous sample drawn from the ear immediately after intubation of the animals. This sample was used as the pre-surgical baseline sample (denoted as PreSx BL). After placement of the pulmonary artery catheter, mixed venous blood samples were taken every 30 min from the first incision until surgery was completed (denoted as S30, S60 etc). The post-surgical baseline sample (denoted as PostSx BL) was designated as time 0, and samples were collected every 30 min after until the commencement of resuscitation (labeled as B0, B30, B60, etc). Blood samples were taken after resuscitation (R) every 30 min (labeled as R30, R60, etc). Blood samples were immediately centrifuged, and plasma was stored at −80°C until further analysis.

### Cytokines and NO_2_^-^/NO_3_^-^ Analysis

TNF-α, IL-10, and IL-6 were measured using human-specific Luminex™ beadsets (BioSource-Invitrogen, Austin, TX) using a Luminex™ 100 IS apparatus (MiraiBio, Austin, TX) or commercially available, pig-specific ELISA kits (R&D Systems, Minneapolis, MN) using a standard ELISA reader. NO_2_^-^/NO_3_^-^ was measured by the nitrate reductase method using a commercially available kit (Cayman Chemical, Ann Arbor, MI).

### Marshall Score (Multiple Organ Dysfunction Score)

Six variables were obtained from the trauma data registry including a) the respiratory system (PO_2_/FIO_2_ ratio); b) the renal system (serum creatinine concentration); c) the hepatic system (serum bilirubin concentration); d) the hematologic system (platelet count); e) the central nervous system (Glasgow Coma Scale) and f) the cardiovascular system (PAR). Marshall Score was calculated according to Marshall JC *et al.* [26].

### Statistical Analysis

Statistical analysis was performed by ANOVA on ranks followed by the Tukey *post hoc* test using Sigma Stat software (Systat Software, San Jose, CA). To compare cytokine levels between survivors and non-survivors in human trauma patients, both Student's *t*-test and the Mann-Whitney rank sum test were applied using Sigma Stat software (Systat Software, San Jose, CA). In all situations in which a statistically significant value is reported (see below), both tests yielded similar results. Correlation between analyte levels and Marshall Scores was assessed by using Spearman's rank correlation coefficient. For all statistical analyses, results are expressed as mean±SEM except when otherwise noted, and a *P* value of <0.05 was considered significant.

## Results

### Inflammation Following Traumatic/Hemorrhagic Shock in Humans

We initially examined the inflammatory responses to T/HS in trauma patients. Twenty-three (19 survivors: 12 males and 7 females, and 4 non-survivors: 3 males and 1 female) human trauma victims of motor vehicle accidents (17) or falls (6) were studied (age: 47±4 yo; ISS: 25±3; ICU LOS: 9±2; total LOS: 13±2; number of days on a ventilator: 4±1). The demographic characteristics of the trauma survivors were the following: age: 43±4 yo; ISS: 25±3; ICU LOS: 9±2; total LOS: 15±3; and number of days on ventilator: 4±1). The demographic characteristics of the trauma non-survivors were the following: age: 66±6 yo (*P* = 0.021); ISS: 24±7; ICU LOS: 8±2; total LOS: 9±2; and number of days on ventilator: 7±2). The non-survivors expired on days 3, 6, 12, and 13 post-admission. The mean Marshall Score in all patients over the time range described above was 3.15±0.5. In survivors, the mean Marshall Score over the same period of time was 2.5±0.45, whereas in non-survivors it was 6±1 (*P* = 0.004).

**Figure 1:**
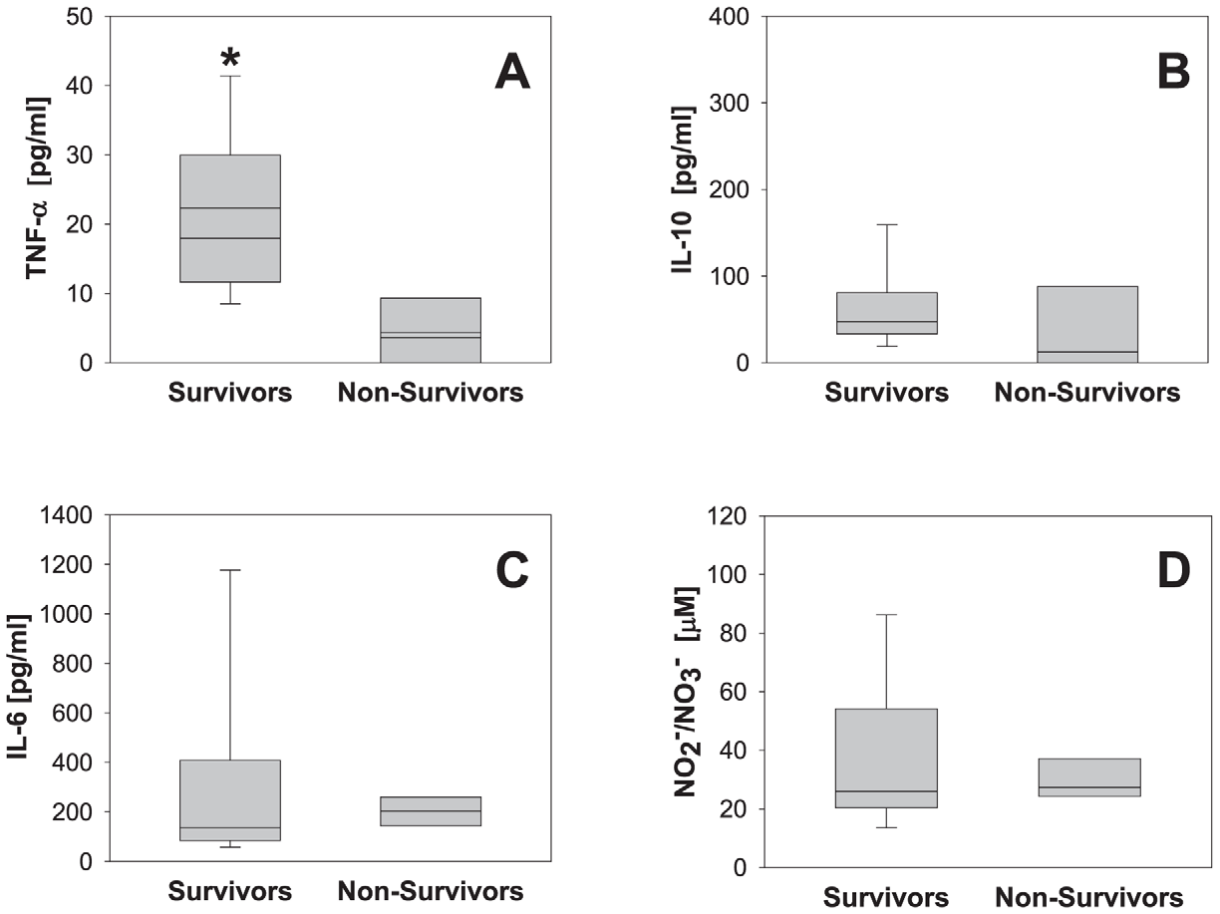
Plasma cytokine and nitrite/nitrate levels in human trauma patients. Plasma samples from 23 patients (19 survivors and 4 non-survivors) taken within the first 6 h following trauma were assayed for TNF-α (A), IL-10 (B), IL-6 (C) and NO_2_^-^/NO_3_^-^ (D) as described in the *Materials and Methods*. Results represent the mean±5^th^ and 95^th^ percentile (*P = 0.025, analyzed by Student's t-test). No statistically significant differences were found in IL-10, IL-6, and NO_2_^-^/NO_3_^-^ levels.

In survivors of polytrauma, the mean levels of cytokines within the first 6 h following trauma were: TNF-α (22±3 pg/ml; range: 7–47 pg/ml), IL-10 (76±18 pg/ml; range: 14–352 pg/ml), and IL-6 (381±151 pg/ml; range: 29–2882 pg/ml). NO_2_^-^/NO_3_^-^ levels were 38±6 µM (range: 12 – 96 µM). In non-survivors, the mean levels of cytokines were: TNF-α (4±3 pg/ml; range: 0–10 pg/ml), IL-10 (33±26 pg/ml; range: 0–109 pg/ml), and IL-6 (202±31 pg/ml; range: 137–265 pg/ml). NO_2_^-^/NO_3_^-^ levels were 30±4 µM (range: 137 – 264 µM) (Fig. 1). A statistically significant difference between survivors and non-survivors was only found for TNF-α (*P* = 0.01; Fig. 1A). In contrast, no statistical significant difference was found for IL-10 (*P* = 0.114; Fig. 1B), IL-6 (*P* = 0.543; Fig. 1C), or NO_2_^-^/NO_3_^-^ (*P* = 0.968; Fig. 1D).

**Figure 2:**
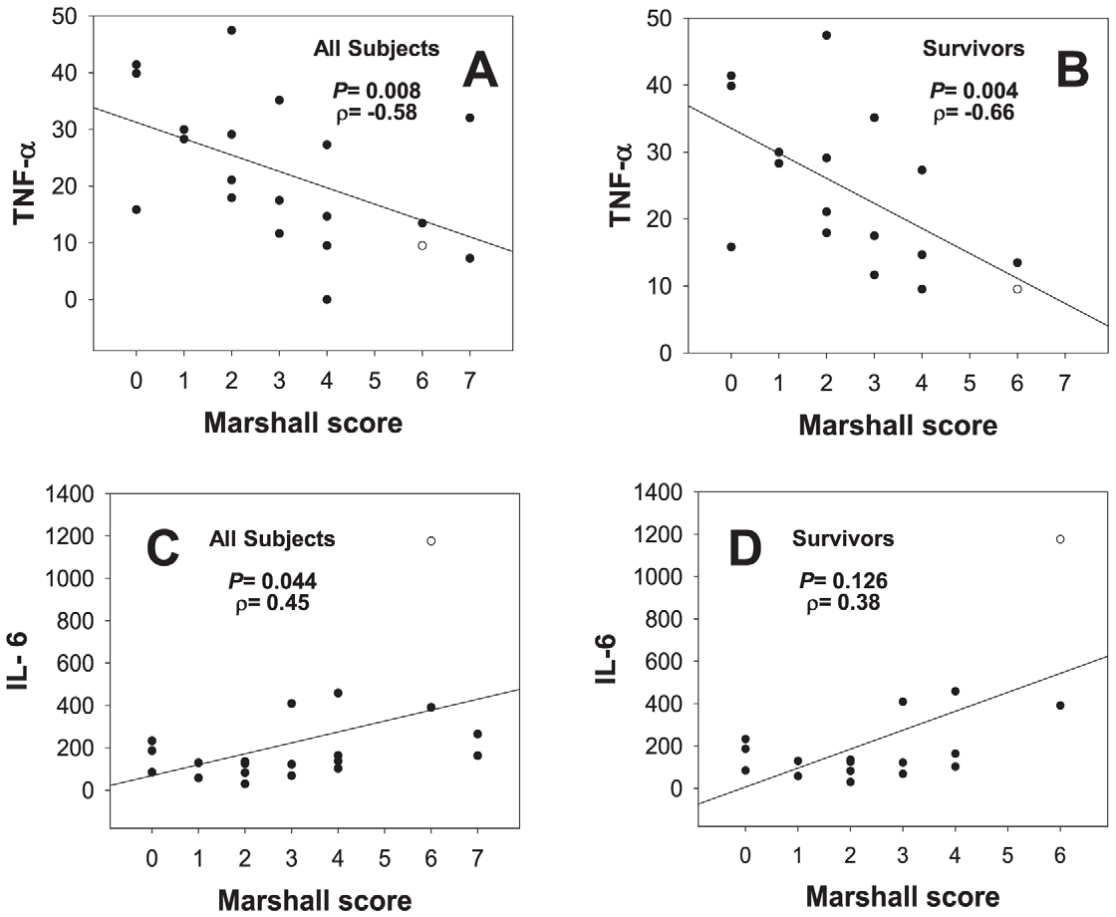
Plasma cytokine levels and Marshall organ damage scores in human trauma patients. Plasma samples from 23 patients (19 survivors and 4 non-survivors) taken within the first 6 h following trauma were assayed for TNF-α (A,B) and IL-6 (C,D) (as described in the *Materials and Methods*) and correlated with Marshall organ damage scores. [(*P<0.05, analyzed by Spearman's rank correlation coefficient (ρ)].

Mortality post-T/HS in humans occurs typically secondary to multiple organ failure [27]. Accordingly, we plotted the levels of TNF, IL-6, IL-10, and NO_2_^-^/NO_3_^-^ as a function of Marshall Score, an index of organ failure, calculated at each blood sampling time point (Fig. 2). We observed a highly significant (*P* = 0.008) negative correlation (correlation coefficient = −0.58) between circulating TNF-α and Marshall Score, taken across all time points in all patients (survivors and non-survivors; Fig. 2A). This finding held true even when examining only survivors (Fig. 2B; *P* = 0.004; correlation coefficient = −0.66). While not directly implying causality, these findings supported our observations regarding a beneficial role of early TNF elevation (Fig. 1A). In contrast, we observed a weaker, but still significant, positive association between circulating IL-6 and Marshall Score taken across all patients (Fig. 2C; *P* = 0.044; correlation coefficient = 0.45). This trend remained but did not reach statistical significance when examining only survivors (Fig. 2D; *P* = 0.126; correlation coefficient = 0.38). No statistically significant associations between inflammatory markers and Marshall Score were observed when examining non-survivors only (data not shown).

These results led us to hypothesize that an early, elevated inflammatory response in the form of the increased TNF-α production was associated with survival. In contrast, we hypothesized that since IL-6 is typically a marker of later inflammation, and indeed may be a hallmark of the positive feedback cycle of inflammation→tissue damage/dysfunction→inflammation, then elevations in this cytokine are indicative of a harmful, self-sustaining inflammatory response. To test these hypotheses, we carried out prospective studies in swine subjected to clinically realistic T/HS.

### Inflammation Following Hemorrhagic Shock in Swine (Group A)

**Figure 3:**
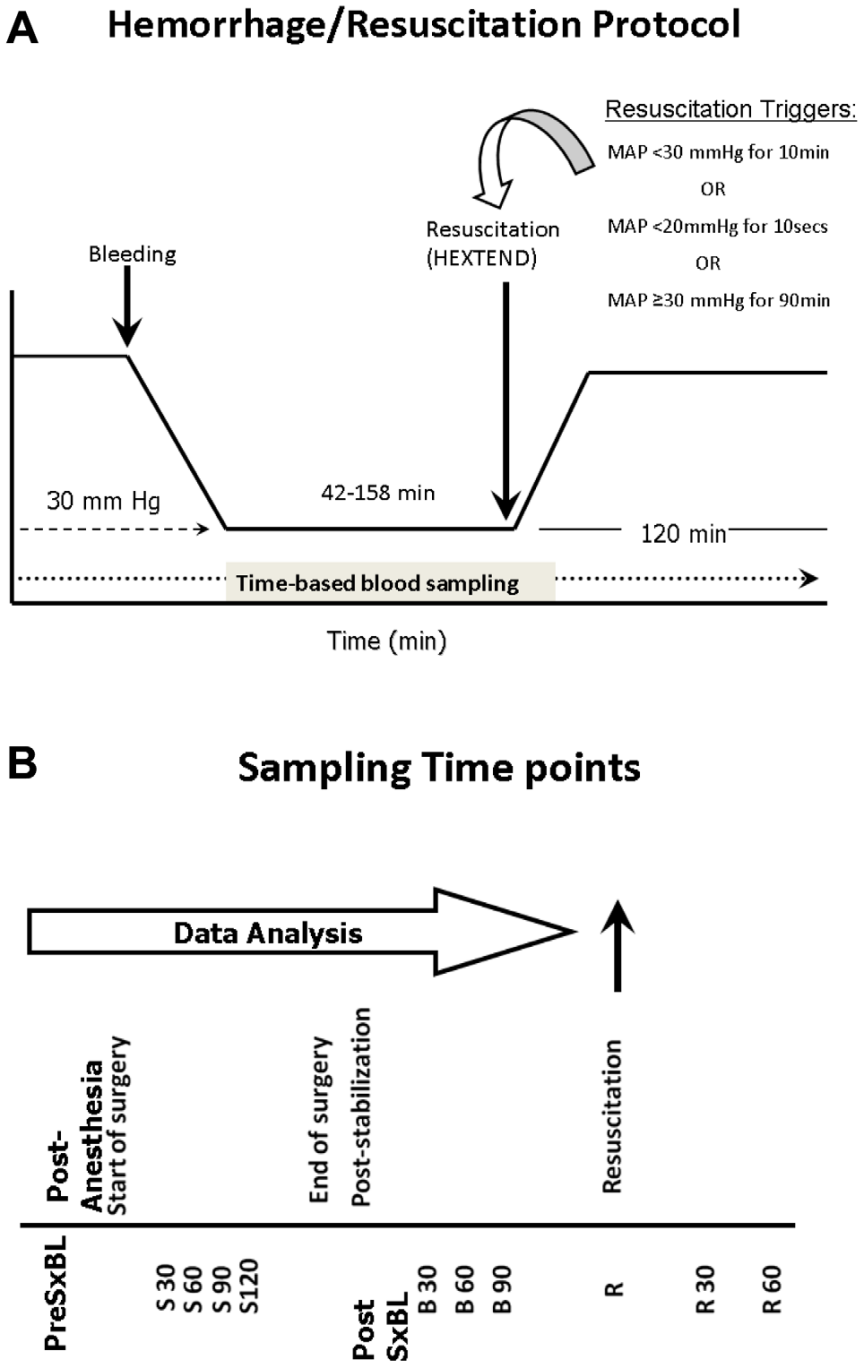
Hemorrhage and sampling protocols for swine T/HS experiments. (A) Hemorrhage protocol. After surgery, animals were stabilized for 15 min. The animals were bled to a MAP of 30 mmHg. Resuscitation was initiated when decompensation occurred, defined by the in the variables in the figure. (B) Blood sampling time points. Every 30 min, blood samples were derived for analysis of TNF-α, IL-10, IL-6, and NO_2_^-^/NO_3_^-^.

A total of 12 pigs (9 survivors and 3 non-survivors) were studied in Group A, following the hemorrhage protocol and hemodynamic parameters that would trigger resuscitation of the animals are depicted in Fig. 3A. Shed blood volume in this group was 882±63 ml (mean±SEM) in survivors and 1063±90 ml in non-survivors. The mean number of bleeds in both survivors and non-survivors was 4, and the mean percent of total volume of blood shed was between 50 and 55 in both survivors and non-survivors. The time to resuscitation in survivors was 113±15 min, whereas in non-survivors it was 132±31 min with no statistical significant difference between time of resuscitation (*P* = 0.557). Given that survival in this experimental paradigm depended in part on intervention by the investigators due to the triggering of defined alarms indicative of hypotension (see Fig. 3A), it was necessary to examine the inflammatory response only up to the point such alarms were triggered, in order to test our hypothesis that robust inflammatory responses were associated with a robust physiological response of the animal to T/HS. No pigs died prior to the last time point utilized in this analysis. The longest time point prior to resuscitation found in common in both survivors and non-survivors was 75 min; accordingly, this was the longest time point utilized in our analyses. Blood sampling was carried out as depicted in Fig. 3B.

**Figure 4:**
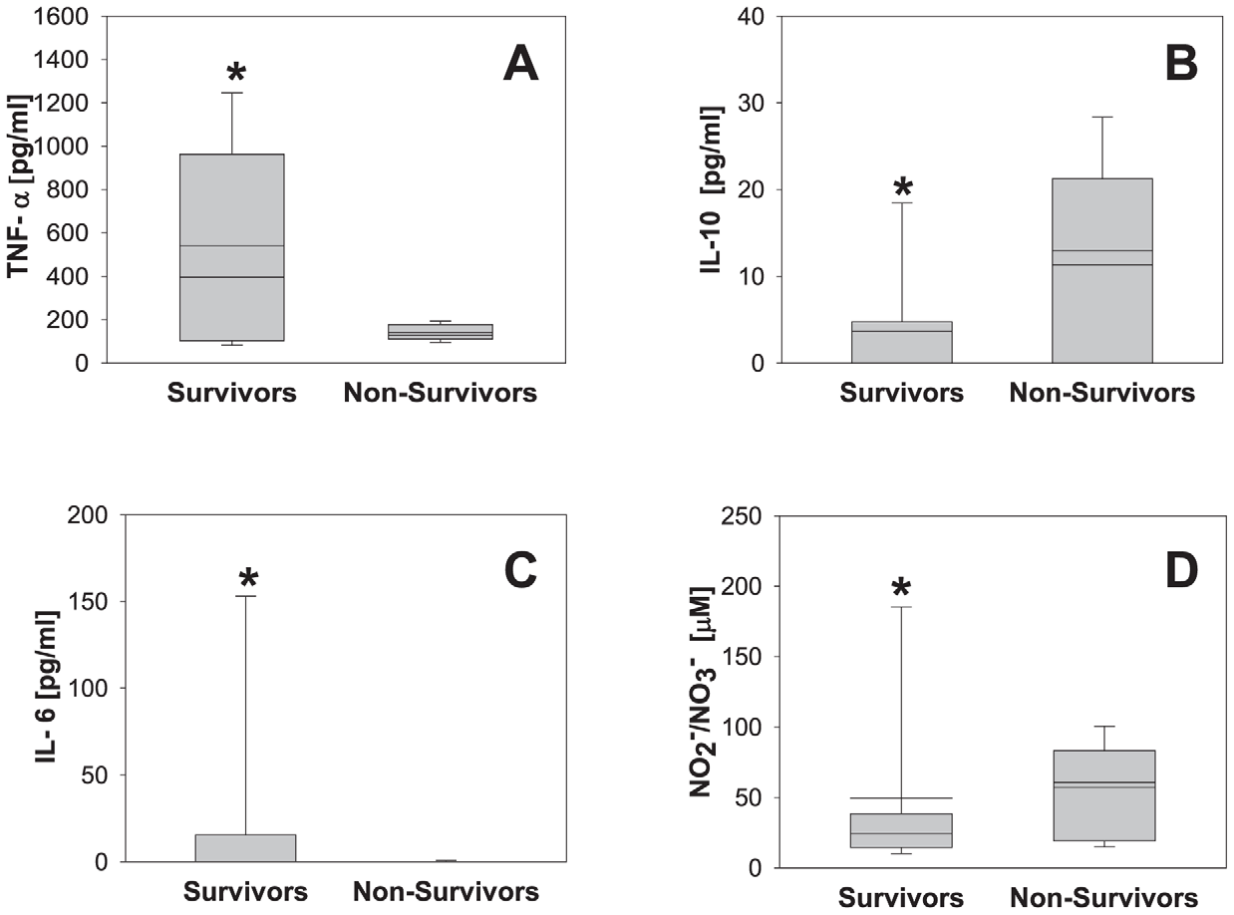
Plasma cytokine and nitrite/nitrate levels in survivors vs. non-survivors in a porcine model of hemorrhagic shock. Plasma samples from 12 pigs (9 survivors and 3 non-survivors) taken at different time points (see Figs. 3A and 5) were assayed for TNF-α (A), IL-10 (B), IL-6 (C) and NO_2_^-^/NO_3_^-^ (D) as described in the *Materials and Methods*. Results represent the mean±5^th^ and 95^th^ percentile (*P<0.05 vs. non-survivors, analyzed by Mann-Whitney Rank Sum Test).

We first examined the levels of TNF-α, IL-10, IL-6, and NO_2_^-^/NO_3_^-^ at all time points in survivors vs. non-survivors (Fig. 4). In survivors, the mean levels of cytokines pre-resuscitation were as follows: TNF-α (540±60 pg/ml; range: 70–1467 pg/ml), IL-10 (3.6±0.9 pg/ml; range: 0–26 pg/ml), and IL-6 (26±8 pg/ml; range: 0–216 pg/ml). NO_2_^-^/NO_3_^-^ levels were 50±8 µM (range: 8–223 µM). In non-survivors, the mean levels of cytokines were as follows: TNF-α (141±8 pg/ml; range: 90–205 pg/ml), IL-10 (11±2 pg/ml; range: 0–35 pg/ml), and IL-6 (0.3±0.2 pg/ml; range: 0–5 pg/ml). NO_2_^-^/NO_3_^-^ levels were 57±7 µM (range: 14–106 µM). Statistically significant differences between survivors and non-survivors were found for TNF-α (*P* = 0.015; Fig. 4A), IL-10 (*P* = 0.003; Fig. 4B), IL-6 (*P* = 0.04; Fig. 4C) and NO_2_^-^/NO_3_^-^ (*P* = 0.043; Fig. 4D).

**Figure 5:**
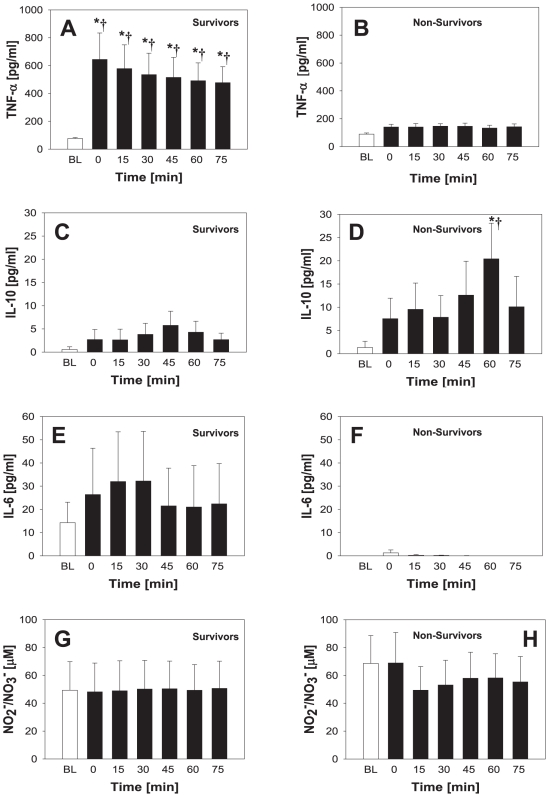
Time course of plasma cytokine and nitrite/nitrate levels in a porcine model of hemorrhagic shock. Plasma samples from 12 pigs (9 survivors and 3 non-survivors) taken at different time points (see Fig. 3A) were assayed for TNF-α (A, B), IL-10 (C, D), IL-6 (E, F) and NO_2_^-^/NO_3_^-^ (G, H) as described in the *Materials and Methods*. Results represent the mean±SEM [*P<0.05 vs. baseline (BL), analyzed by One-Way ANOVA followed by the Tukey *post hoc* test]. The TNF-α levels (0–75 min) in survivors (A) were also statistically different from the levels measured in non-survivors (B), indicated by †. The IL-10 levels (at 0, 15 and 60 min) in non-survivors (D) were statistically different from the levels measured in survivors (C), indicated by †. BL: baseline.

We next examined the levels of inflammatory analytes as a function of time in survivors vs. non-survivors. A statistically significant difference in TNF-α was found between survivors and non-survivors at several experimental time points (15 min post-surgical baseline; at the start of hemorrhage [0]; and at 15, 30, 45, 60, and 75 min post-hemorrhage; Fig. 5A). A statistically significant difference in circulating IL-10 was observed between survivors and non-survivors at 15 min post-surgical baseline as well as at 15 and 60 min post-hemorrhage (Fig. 5D). No statistically significant difference was found in either IL-6 or NO_2_^-^/NO_3_^-^ levels between survivors and non-survivors (*Supplementary Materials*, Table S1).

We next examined the trends in cytokines within groups. In survivors, mean serum TNF-α at baseline was 77±7 pg/ml, rose in a statistically significant fashion following the initial surgery, and remained elevated up to 75 min post-hemorrhage (with statistically significant changes vs. baseline at 0, 15, 30, 45, 60, and 75 min post-hemorrhage; Fig. 5A). In contrast, while the mean baseline TNF-α value in non-survivors was similar to that of the survivors (89±9 pg/ml *vs.* baseline TNF-α in survivors), no statistically significant change vs. baseline occurred either following surgery or during the hemorrhage period (Fig. 5B). We observed essentially the opposite behavior for IL-10: there was no significant change in IL-10 in survivors from baseline (1±0.6 pg/ml) which did not change over time (at 75 min was 3±1.4 pg/ml; Fig. 5C), while non-survivors had a baseline IL-10 value of 1±1.3 pg/ml, which rose to 20±7.7 pg/ml (with a significant change at 60 min vs. pre-operative baseline; Fig. 5D). The levels of IL-6 and NO_2_^-^/NO_3_^-^ were uniformly low and unchanged in both survivors (Figs. 5E and 5G, respectively) and non-survivors (Figs. 5F and 5H, respectively; approximately 24 pg/ml IL-6 and 50 µM NO_2_^-^/NO_3_^-^ vs. 0 pg/ml IL-6 and 59 µM NO_2_^-^/NO_3_^-^, respectively) throughout the 75 min time course analyzed in this study.

The control group (surgery only) for Group A (*Supplementary Materials*, Fig. S1 and Table S2) exhibited low mean pre-operative baseline levels of all cytokines and NO_2_^-^/NO_3_^-^, that remained low throughout the observation time period (5.5 h). The levels of these inflammatory analytes were as follows: TNF-α (mean levels pre-operative baseline 75±7 pg/ml vs. 75±17 pg/ml at the end; Fig. S1A), IL-10 (12±7 pg/ml at baseline vs. 14±2 pg/ml at the end; Fig. S1B), IL-6 (below detection limit at baseline vs. 5±5 pg/ml at the end; Fig. S1C), and NO_2_^-^/NO_3_^-^ (37±9 µM vs. 24±5 µM at the end; Fig. S1D).

The results observed in the T/HS pigs supported the hypothesis, derived from the observation of human trauma patients, that early elevation in TNF-α following substantial injury was associated with survival. These results further suggested that in settings in which injury was below a certain threshold (i.e., in the swine subjected to minor cannulation surgery only), only mild inflammation was induced. To further test these hypotheses, we sought to carry out additional studies in swine subjected to a larger degree of T/HS.

### Inflammation Following Hemorrhagic Shock Pigs Combined with Anterolateral Thoracotomy (Group B)

A total of 11 (7 survivors and 4 non-survivors) swine were studied in Group B (Fig. 5); as for Group A, the hemorrhage protocol is depicted in Fig. 3A and the blood sampling protocol is depicted in Fig. 3B. Shed blood volume in this group was 700±48 ml in survivors and 662±138 ml in non-survivors. The mean number of bleeds in both survivors and non-survivors was 3, and the mean percent of total volume of blood shed was between 20 and 22 in both survivors and non-survivors. The time to resuscitation in survivors was 129±6 min, whereas in non-survivors it was 99±19 min, with no statistically significant difference between resuscitation time (*P* = 0.093). The longest time point prior to resuscitation found in common in both survivors and non-survivors was 90 min. Utilizing the same method of analysis as in Group A, 90 min was therefore the longest time point utilized for all subsequent analyses.

**Figure 6:**
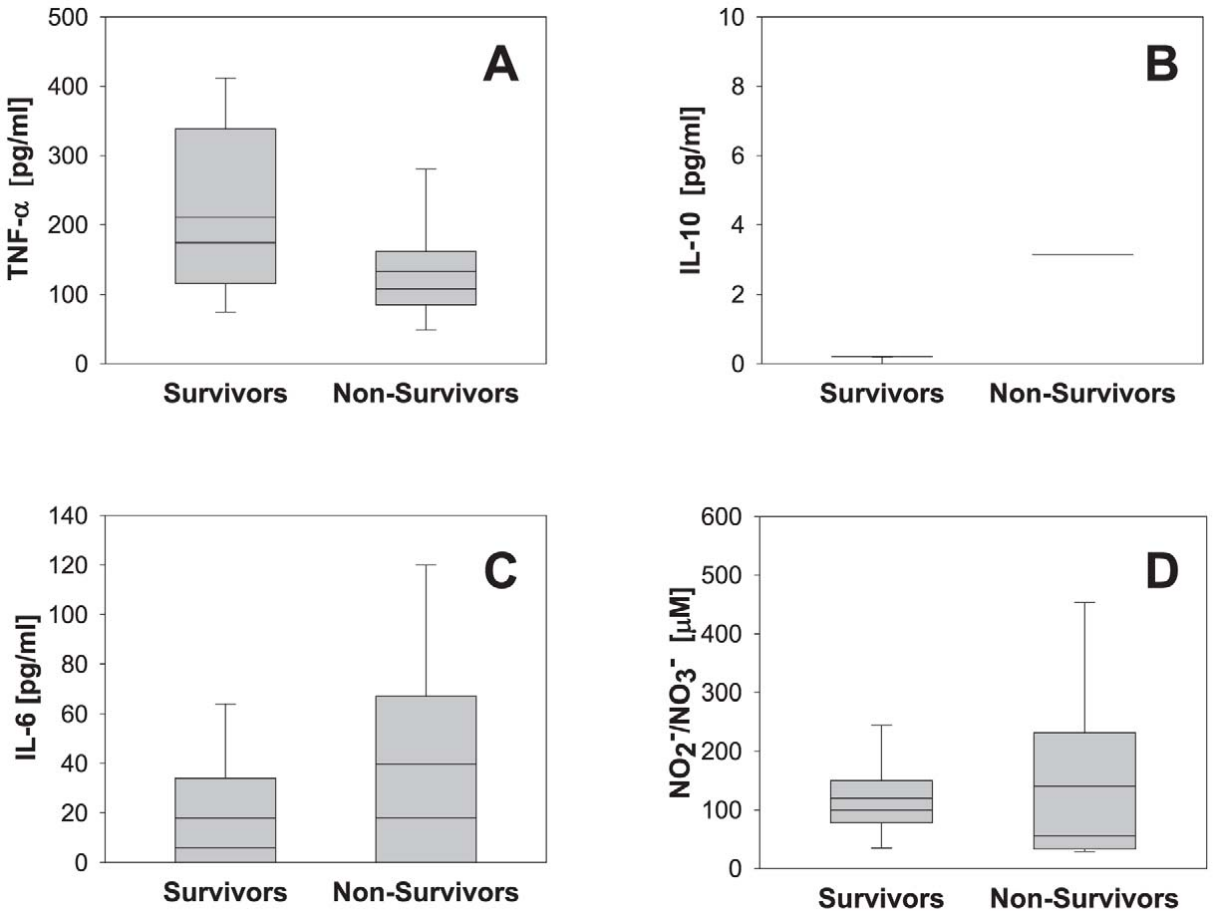
Plasma cytokine and nitrite/nitrate levels in survivos vs. non-survivors in a swine model of anterolateral thoracotomy + hemorrhagic shock. Plasma samples from 11 swine (7 survivors and 4 non-survivors) taken at different time points (see Figs. 3A and 7) were assayed for TNF-α (A), IL-10 (B), IL-6 (C) and NO_2_^-^/NO_3_^-^ (D) as described in the *Materials and Methods*. Results represent the mean±5^th^ and 95^th^ percentile.

As for Group A, we first examined the levels of TNF-α, IL-10, IL-6, and NO_2_^-^/NO_3_^-^ at all time points in survivors vs. non-survivors (Fig. 6). In survivors, the mean levels of cytokines pre-resuscitation were as follows: TNF-α (210±23 pg/ml; range: 60–449 pg/ml), IL-10 (0.2±0.1 pg/ml; range: 0–3.8 pg/ml), and IL-6 (18±94 pg/ml; range: 0–87 pg/ml). NO_2_^-^/NO_3_^-^ levels were 119±11 µM (range: 27–264 µM). In non-survivors, the mean levels of cytokines were as follows: TNF-α (144±20 pg/ml; range: 48–350 pg/ml), IL-10 (3.15±3 pg/ml; range: 0–63 pg/ml), and IL-6 (40±12 pg/ml; range: 0–209 pg/ml). NO_2_^-^/NO_3_^-^ levels were 140±37 µM (range: 28–477 µM). No statistically significant differences in these analytes were found between survivors and non-survivors, however. Interestingly, IL-10 levels in the non-survivors in Group B were significantly lower than those of the non-survivors in Group A. In contrast, Group B IL-6 levels were significantly higher in non-survivors vs. IL-6 levels in Group A non-survivors. Finally, the NO_2_^-^/NO_3_^-^ levels of Group B survivors were significantly higher than the NO_2_^-^/NO_3_^-^ levels of Group A survivors (all cross-group comparisons were performed using the Kruskal-Wallis One Way Analysis of Variance on Ranks, with significance determined at *P*<0.05).

**Figure 7:**
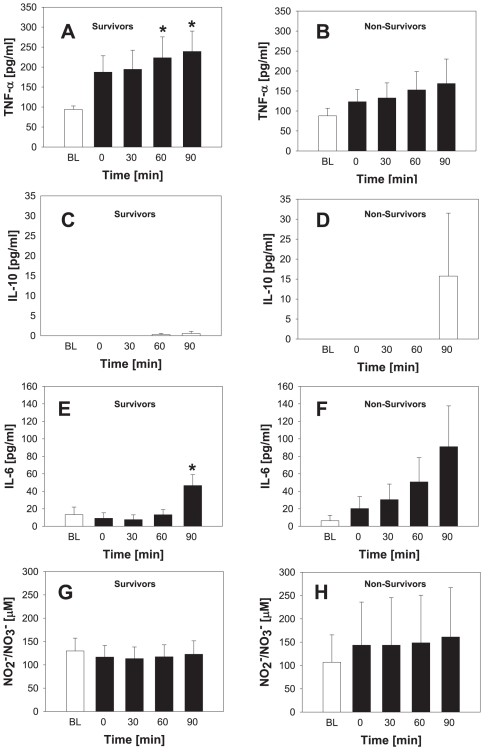
Time course of plasma cytokine and nitrite/nitrate levels in a porcine model of hemorrhagic in combination with anterolateral thoracotomy. Plasma samples from 11 swine (7 survivors and 4 non-survivors) taken at 0, 30, 60 and 90 min (see Fig. 3A) were assayed for TNF-α (A, B), IL-10 (C, D), IL-6 (E, F) and NO_2_^-^/NO_3_^-^ (G, H) as described in the *Materials and Methods*. Results represent the mean±SEM [*P<0.05 vs. baseline (BL), analyzed by One-Way ANOVA followed by the Tukey *post hoc* test]. BL: baseline.

We next examined the trends in cytokines within groups. In survivors, the mean serum TNF-α at baseline was 94±9 pg/ml. Statistically significant elevations vs. baseline in TNF-α were observed at 60 min (223±52 pg/ml) and 90 min (239±51 pg/ml) post-hemorrhage (Fig. 7A). In contrast, and similarly to Group A, the mean baseline TNF-α value in non-survivors was similar to that of the survivors (88±19 pg/ml), and no statistically significant change occurred either following surgery or during the hemorrhage period (Fig. 7B). We observed no statistically significant change in IL-10 from baseline in either survivors (Fig. 7C) or non-survivors (Fig. 7D) at any time point post-surgery. IL-6 levels were statistically significantly different at 90 min vs. preoperative baseline only in survivors (*P* = 0.02; Fig. 7E). The levels NO_2_^-^/NO_3_^-^ (Figs. 7G-7H) were uniformly low in both survivors and non-survivors at baseline, and did not change significantly over the time course analyzed.

As in Group A, the control group (surgery only) for Group B (*Supplementary Materials*, Fig. S2 and Table S2) exhibited similar pre-operative baseline mean levels of all cytokines and NO_2_^-^/NO_3_^-^, that remained low throughout the observation time period (5 h). The levels of these inflammatory analytes were as follows: TNF-α (mean levels pre-operative baseline 82±5 pg/ml vs. 86±10 pg/ml at the end; Fig. S2A), IL-10 (below detection limit at baseline as well as at the end; Fig. S2B), IL-6 (below detection limit at baseline vs. 4±4 pg/ml at the end; Fig. S2C), and NO_2_^-^/NO_3_^-^ (63±21 µM vs. 53±21 µM at the end; Fig. S2D). Taken together, these results supported the hypothesis that early inflammation was associated with survival, and that sub-threshold, survivable injury did not induce substantial inflammation.

## Discussion

The biology of inflammatory cytokines and related products such as NO is highly complex [8], [9], and especially so in very acute processes such as the response to T/HS [7], [10], [11]. This complexity is not surprising. Inflammation may be considered as a communication network that must integrate various stimuli and orchestrate an appropriate set of responses to these stimuli, in a manner that depends on the nature of the initiating stimulus and host-related factors (i.e. gender, age, and genetics). The acute inflammatory response is integrated with various physiological systems, an interaction that in the setting of T/HS acts both as a means of relaying information about the nature of the insult and as an outcome (e.g. degree of decompensation). Due to this complexity, extreme care must be taken when attempting to use cytokines as diagnostic and prognostic markers of outcome following T/HS [28], especially so if cytokines are to be future therapeutic alternatives [29].

Our primary goal in the present study was to examine a central question relating to the role of cytokines in post-T/HS inflammation: is early, robust inflammation–whose hallmark is the elevation of inflammatory cytokines such as TNF-α–associated with benefit or detriment? More specifically, we sought to associate the early inflammatory response of swine to T/HS with 1) the need for resuscitation and 2) response to later resuscitation (survival). In parallel, we sought to determine if the phenomena observed in the setting of experimental T/HS also held true in human T/HS.

Our studies suggest that a robust, early TNF-α response is associated with survival in trauma victims, a finding supported by data in large experimental animals subjected to T/HS. Indeed, the trauma patient cohort examined, both survivors and non-survivors, exhibited an inverse correlation between TNF-α production and organ damage/dysfunction. Moreover, this inverse correlation was observed even within the survivor sub-group, suggesting that early TNF-α serve either to limit organ damage or to induce reparative processes. While elevated plasma levels of TNF-α have been found in both hemorrhagic shock patients [30]–[32] and in experimental animal models [33]–[35], our results suggest that this early, alarm-phase cytokine may need to be recast as being beneficial when indicating a self-limiting form of inflammation that signals for healing of injury.

The concept that inflammation is beneficial post-trauma may, at first glance, appear to contradict a large body of literature that points to morbidity and mortality associated with elevated inflammatory cytokines post-T/HS. However, attempts at modulating the canonical early pro-inflammatory cytokine TNF-α in the setting of T/HS have had mixed results. Bemelmans *et al.* found that administering anti-TNF-α antibodies to jaundiced mice subjected to surgical trauma was not associated with improvement in survival [36]. Similarly, mortality of wild-type mice subjected to hemorrhagic shock was unaffected by pre-treatment with anti-TNF-α antibodies [37]. In contrast, Zingarelli *et al.* found that ant-TNF-α antibodies improved survival in an extremely severe paradigm of hemorrhagic shock in rats (death by 30 minutes post-hemorrhage) [38]. Various studies suggested improvements in histological parameters following treatment with anti-TNF-α in the settings of T/HS, but did not document effects on survival. For example, Marzi *et al.* found that anti-TNF-α antibodies attenuated leukocyte adhesion in the livers of rats subjected to HS [39], and Abraham *et al.* found evidence of reduced lung inflammation [40].

Indeed, a closer look suggests that the primary elevated inflammatory cytokine is IL-6, which we have suggested through computational studies may be indicative of a positive feedback loop of inflammation→tissue damage/dysfunction→inflammation [15] Interleukin-6 is arguably the best biomarker of outcome of trauma patients with Systemic Inflammatory Response Syndrome, sepsis, and Multiple Organ Failure [13], [25]. Though we observed a weak, positive correlation between circulating IL-6 and Marshall Score, we did not observe any significantly elevated levels of IL-6 in either human or porcine T/HS. Ayala *et al.* found that IL-6 increased continuously post-hemorrhage and was already increased after midline laparotomy and before initiation of hemorrhage compared with non-manipulated animals, while TNF-α was only detected once hemorrhage was initiated [35], [41]. These studies suggested that soft tissue trauma might be a potent stimulus to the production of IL-6 [41]. Our experiments comparing hemorrhaged animals to their surgery-only controls support these studies, but suggest that there is a threshold for overall injury that must be exceeded before TNF-α elevations are observed in the circulation.

An evolving literature points to a central role for the release of “alarm/danger” signals (also known as “Damage-Associated Molecular Pattern” molecules) which may damage tissues and cause the dysfunction of organs, and re-induce the release of TNF-α in a vicious cycle [42], [43]. In the present study, we found a positive association between IL-6 and organ dysfunction in trauma patients, as well as elevated plasma IL-6 levels after 60 min of hemorrhage in swine, consistent with this notion. The levels of IL-6 have been repeatedly reported to be elevated in both animal and clinical studies of T/HS [35], [41], [44]–[47]. We note that in our relatively short-term animal study, there was no difference in IL-6 levels between survivors and non-survivors; additionally, the relationship between early IL-6 levels and late complications after trauma and hemorrhage was not studied. Our results therefore suggest that, as in contrast to sepsis [48], early elevations of IL-6 may play a prominent role in the response to T/HS. Later elevations in IL-6 are also associated with morbidity [49], [50], and thus persistent elevations in IL-6 may be indicative of self-sustaining, tissue-damaging inflammation.

T_H_2 cytokines, central among them IL-10, are thought to contribute to immunosuppression and the development of sepsis [7]. IL-10, which is characterized as an anti-inflammatory cytokine [23], [31], [51], [52] was assessed in T/HS patients [53] and found to be a potent down-regulator of cell-mediated immune and pro-inflammatory responses [54], [55]. Experimental studies have demonstrated that IL-10 inhibits the production of pro-inflammatory cytokines, such as TNF-α and IL-6, by activated macrophages [54]–[57]. Moreover, it has been demonstrated that IL-10 is an immunosuppressant in animal models of T/HS [58]. We suggest that at early time points following trauma, circulatory and the neuro-endocrine derangements lead to the production of catecholamines, which in turn induce this later production of IL-10 [29], [33], [59], [60]. Overly elevated IL-10 could suppress TNF-α produced by monocyte/macrophages in multiple tissues [56], perhaps accounting for the cytokine phenotype we have observed in non-survivor pigs. Investigators have suggested the possibility of gene therapy with IL-10 for acute inflammatory syndromes such as T/HS [61]–[64]; our studies suggest that caution should be exercised when considering intervention using this cytokine.

Finally, since NO contributes to the host's inflammatory defense and can cause circulatory disorders, it may be an important mediator in the setting of inflammation and organ failure, possibly by altering outcome after T/HS [13]. Endothelial cells produce NO from a largely constitutive isoform of NO synthase (NOS), whose expression and activity is known to be reduced in hemorrhagic shock [65]–[67]. Most cells also produce NO via an inducible form of NOS (iNOS), the expression of which is induced by cytokines [68] Elevated NO_2_^-^/NO_3_^-^ levels in trauma patients have been previously reported both immediately after trauma [69] and at later time points [70]. This elevated NO production reflected severity of injury during the first two hours after the traumatic insult, suggesting that increased NO production might play a role in the very early post-injury period [71]. Other studies have focused on NO as a possible mediator of decompensation, with increases in iNOS activity being reported in several organs after prolonged hemorrhagic shock [72].

In the present study, we observed significant differences between survivors and non-survivors only in Group A swine only when the NO_2_^-^/NO_3_^-^ data were taken as a whole for each outcome group, but not in trauma patients and not as a function of time in injured swine. These results suggest that iNOS is probably not involved in the phenomena studied herein, though they may suggest that eNOS activity is altered in some settings (consistent with prior findings [65]–[67]). The lack of a role for iNOS in swine may reflect the early time points studied. Alternatively, the activity of eNOS might be affected differentially in T/HS survivors vs. non-survivors, but the relative insensitivity of NO_2_^-^/NO_3_^-^ as a measurement outcome may necessitate alternative methods (e.g. directly measuring NO by a NO-sensitive electrode or other means [73]) to address this point.

A general limitation of our study centers on the fact that the overall number of subjects and inflammatory analytes studied was relatively low in both humans and swine. With regard to patient number, we believe that our data (17% mortality rate) are representative of the type of outcomes seen in patients presenting with blunt trauma (e.g. the stud of Sperry *et al.* [49], in which only 5% mortality is reported in trauma patients). Given this limitation, we augmented our study by assaying inflammatory analytes in serial samples from trauma patients. We also carried out a clinically realistic animal model of trauma/hemorrhage, in which defined alarms triggered resuscitation and led to a low mortality that is concordant with that seen in trauma patients. We believe that our results are valid because even when examining all samples from all patients (both survivors and non-survivors) we found a negative correlation between early circulating TNF-α levels and organ damage, suggesting that an early pro-inflammatory response is associated with a positive outcome. In the same cohort, circulating IL-6 correlated positively with organ damage, as would be expected from a large number of studies that have examined circulating IL-6 in trauma patients (thereby helping validate, at least in part, the cohort of patients studied herein). The choice of cytokines utilized in the analysis described in the present manuscript was based on a defined number of cytokines that have been well-vetted with regard to their role in trauma/hemorrhage (as described above) and that could be measured in both humans and swine. We hope that as the number of available pig-specific cytokine assay kits increases, we will be able to expand the present study to a broader panel of inflammatory mediators.

Another caveat that needs to be considered with regard to our studies in swine concerns the experimental protocol used. Our model of severe hemorrhagic shock allows for a wide range of hemodynamic fluctuations in the course of the experiment and reflects the animals' compensatory responses. Not surprisingly, this experimental paradigm was associated with significant inter-animal variability in both time course and outcome. Furthermore, analysis was carried out up only to the pre-resuscitation time point due to the fact that resuscitation has been shown to influence the inflammatory response to trauma and hemorrhage [74]. Ideally, swine should be subjected to a combination of soft tissue injury and bone fracture, in combination with mild to moderate hemorrhagic shock, in order to simulate the types of blunt injury seen in trauma patients. However, due to limitations placed on us by animal use regulations, we cannot easily carry out such studies, and moreover could not carry out such studies and then recover the animals and follow them for 1–2 days. Nonetheless, we suggest that the characteristics of our experimental preparation render it particularly suitable for the practical assessment of dynamic response characteristics, and especially since one of our central goals was to compare the responses of large experimental animals with those of human trauma victims.

Alternative hypotheses may be raised with regard to our findings. Higher circulating levels of TNF, IL-6, IL-10, and NO_2_^-^/NO_3_^-^ are found in septic patients [70], [75]–[77]. In one study, higher levels of plasma cytokines were reported in non-survivors of sepsis, and in this study fluid resuscitation was associated with lower mean cytokine levels [77]. Rivers *et al.* have hypothesized that better perfused organs suffer less damage/dysfunction, and thus are less inflamed [78]. Thus, it may be argued that trauma results in such profound hypoperfusion that cytokines are not flushed out of damaged organs, contrary to sepsis, where many organs remain perfused. This alternative hypothesis could be tested (at least in experimental animals) by quantifying tissue levels of cytokines.

Another limitation of our comparative study in humans and swine involves a somewhat different sampling methodology in these two species; clearly, we were able to sample blood in experimental animals more frequently than in trauma patients. Despite the fact the time points in both settings fell within a 6-h range, it may be argued that we are observing different kinetics of cytokine production and therefore different phenomena.

In conclusion, our studies suggest that the role of TNF-α in T/HS may need to be re-evaluated in light of our findings. On a broader level, there may be a need to distinguish between early and late inflammation induced by injury. Our studies suggest that early, adequately robust production of TNF-α following injury is a hallmark of a proper response, while unchanging, low-levels of this cytokine may reflect pathology. This situation may be analogous to that observed when studying physiologically variable responses such as heart rate [79]–[81]. Though further study is warranted, our findings raise the possibility of re-interpreting the role of TNF-α post-T/HS, and suggest that caution should exercised when thinking of TNF-α antagonism in this setting.

## Supporting Information

Table S1 Circulating cytokine levels in swine subjected to experimental T/HS. See Materials and Methods for details. HS = Hemorrhagic Shock, HS + T = Hemorrhagic Shock + Thoracotomy, n = number of animals/condition.
(0.07 MB DOC)

Table S2 Circulating cytokine levels in swine subjected to experimental Surgery and Surgery with Thoracotomy. See Materials and Methods for details. n = number of animals/condition.
(0.08 MB DOC)

Figure S1 Plasma cytokine and nitrite/nitrate levels in pigs subjected to surgical cannulation only. Plasma samples from 3 swine (all survivors) taken at different time points (see Fig. 2B) were assayed for TNF-α (A), IL-10 (B), IL-6 (C) and NO_2_^-^/NO_3_^-^ (D) as described in the *Materials and Methods*. Results represent the mean±SEM (*P<0.05 vs. baseline, analyzed by One-Way ANOVA followed by the Tukey *post hoc test*).
(0.19 MB TIF)

Figure S2 Plasma cytokine and nitrite/nitrate levels in pigs subjected to surgical cannulation in combination with anterolateral thoracotomy only. Plasma samples from 4 swine (all survivors) taken at different time points (see Fig. 2B) were assayed for TNF-α (A), IL-10 (B), IL-6 (C) and NO_2_^-^/NO_3_^-^ (D) as described in the *Materials and Methods*. Results represent the mean±SEM (*P<0.05 vs. baseline, analyzed by One-Way ANOVA followed by the Tukey *post hoc test*).
(0.19 MB TIF)
