# Supplementary material for: An Adequately Robust Early TNF-α Response Is a Hallmark of Survival Following Trauma/Hemorrhage
Source: PLoS One. 2009 Dec 22;4(12):e8406. doi: 10.1371/journal.pone.0008406 (PMC2794373; doi:10.1371/journal.pone.0008406)
Supplement: Table S1 — Circulating cytokine levels in swine subjected to experimental T/HS. See Materials and Methods for details. HS = Hemorrhagic Shock, HS + T = Hemorrhagic Shock + Thoracotomy, n = number of animals/condition. (0.07 MB DOC) [file pone.0008406.s001.doc]

**Table S1: Circulating cytokine levels in swine subjected to experimental T/HS.** See *Materials and Methods* for details. HS = Hemorrhagic Shock, HS + T = Hemorrhagic Shock + Thoracotomy, n = number of animals/condition.

|  | **TNF-α** | | | |
| --- | --- | --- | --- | --- |
|  | Survivors | | Non Survivors | |
| **Time** | **HS (n=9 )** | **HS+T (n=7 )** | **HS (n=3 )** | **HS+T (n=4)** |
| **Baseline** | 77 ± 7 | 94 ± 9 | 89 ± 9 | 88 ± 19 |
| **0** | 644 ± 190 | 187 ± 41 | 141 ± 19 | 123 ± 31 |
| **15** | 578 ± 171 | - | 141 ± 25 | - |
| **30** | 535 ±1 54 | 194 ± 48 | 146 ± 18 | 133 ± 38 |
| **45** | 515 ± 143 | - | 146 ± 23 |  |
| **60** | 491 ± 128 | 223 ± 52 | 133 ± 20 | 153 ± 46 |
| **75** | 478 ± 114 | - | 142 ± 21 | - |
| **90** | - | 239 ± 51 | - | 169 ± 61 |
|  |  |  |  |  |
|  | **IL-10** | | | |
|  | Survivors | | Non Survivors | |
| **Time** | **HS (n=9)** | **HS+T (n=7)** | **HS (n=3)** | **HS+T (n=4)** |
| **Baseline** | 1 ± 1 | 0 ± 0 | 1 ± 1 | 0 ± 0 |
| **0** | 3 ± 2 | 0 ± 0 | 8 ± 4 | 0 ± 0 |
| **15** | 3 ± 2 | - | 10 ± 6 | - |
| **30** | 4 ± 2 | 0 ± 0 | 8 ± 5 | 0 ± 0 |
| **45** | 6 ± 3 | - | 13 ± 7 | - |
| **60** | 4 ± 2 | 0 ± 0 | 20 ± 8 | 0 ± 0 |
| **75** | 3 ± 1 | - | 10 ± 7 | - |
| **90** | - | 1 ± 1 | - | 16 ± 16 |

|  | **IL-6** | | | |
| --- | --- | --- | --- | --- |
|  | Survivors | | Non Survivors | |
| **Time** | **HS (n=9 )** | **HS+T (n=7 )** | **HS (n=3 )** | **HS+T (n=4 )** |
| **Baseline** | 14 ± 9 | 13 ± 9 | 0 ± 0 | 6 ± 6 |
| **0** | 26 ± 20 | 9 ± 6 | 1 ± 1 | 20 ± 14 |
| **15** | 32 ± 21 | - | 0 ± 0 | - |
| **30** | 32 ± 21 | 8 ± 5 | 0 ± 0 | 31 ± 18 |
| **45** | 21 ± 16 | - | 0 ± 0 | - |
| **60** | 21 ± 18 | 13 ± 6 | 0 ± 0 | 51 ± 28 |
| **75** | 22 ± 17 | - | 0 ± 0 | - |
| **90** | - | 47 ± 12 |  | 91 ± 47 |
|  |  |  |  |  |
|  | **NO2^-^/ NO3^-^** | | | |
|  | Survivors | | Non Survivors | |
| **Time** | **HS(n=9)** | **HS+T (n=7)** | **HS (n=3)** | **HS+T (n=4 )** |
| **Baseline** | 49 ± 20 | 130 ± 28 | 69 ± 20 | 107 ± 59 |
| **0** | 48 ± 21 | 117± 25 | 69 ± 22 | 144 ± 92 |
| **15** | 49 ± 22 |  | 49 ± 17 |  |
| **30** | 50 ± 21 | 113 ± 25 | 53 ± 18 | 144 ± 102 |
| **45** | 50 ± 20 | - | 58 ± 19 |  |
| **60** | 49 ± 18 | 117 ± 26 | 58 ± 17 | 149 ± 102 |
| **75** | 51 ± 20 | - | 55 ± 18 | - |
| **90** | - | 123 ± 29 | - | 161 ± 106 |
