# Supplementary material for: An Adequately Robust Early TNF-α Response Is a Hallmark of Survival Following Trauma/Hemorrhage
Source: PLoS One. 2009 Dec 22;4(12):e8406. doi: 10.1371/journal.pone.0008406 (PMC2794373; doi:10.1371/journal.pone.0008406)
Supplement: Table S2 — Circulating cytokine levels in swine subjected to experimental Surgery and Surgery with Thoracotomy. See Materials and Methods for details. n = number of animals/condition. (0.08 MB DOC) [file pone.0008406.s002.doc]

**Table S2: Circulating cytokine levels in swine subjected to experimental Surgery and Surgery with Thoracotomy.** See *Materials and Methods* for details. n= number of animals/condition.

|  | **TNF-α** | |  |  | **IL-10** | |
| --- | --- | --- | --- | --- | --- | --- |
|  | Surgery only | Surgery +Thoracotomy |  |  | Surgery only | Surgery +Thoracotomy |
| **Time** | **n=3** | **n=4** |  | **Time** | **n=3** | **n=4** |
| **Baseline** | 75 ± 7 | 82 ± 5 |  | **Baseline** | 12 ± 7 | 0 ± 0 |
| **0** | 72 ± 6 | 90 ± 6 |  | **0** | 11 ± 6 | 1 ± 1 |
| **30** | 61 ± 7 | 86 ± 3 |  | **30** | 10 ± 5 | 3 ± 3 |
| **60** | 81 ± 22 | 92 ± 6 |  | **60** | 6 ± 4 | 0 ± 0 |
| **90** | 78 ± 18 | 93 ± 8 |  | **90** | 13 ± 7 | 0 ± 0 |
| **120** | 85 ± 20 | 89 ± 8 |  | **120** | 8 ± 4 | 2 ± 2 |
| **150** | 89 ± 22 | 94 ± 6 |  | **150** | 22 ± 13 | 0 ± 0 |
| **180** | 70 ± 11 | 93 ± 7 |  | **180** | 21 ± 16 | 0 ± 0 |
| **210** | 68 ± 13 | 91 ± 6 |  | **210** | 10 ± 8 | 0 ± 0 |
| **240** | 77 ± 18 | 83 ± 7 |  | **240** | 9 ± 5 | 0 ± 0 |
| **270** | 72 ± 15 | 79 ± 10 |  | **270** | 12 ± 2 | 0 ± 0 |
| **300** | 67 ±12 | 86 ±10 |  | **300** | 17 ±9 | 0 ±0 |
| **330** | 75 ±17 | - |  | **330** | 14 ±2 | - |
|  |  |  |  |  |  |  |
|  |  |  |  |  |  |  |
|  | **IL-6** | |  |  | **NO2^-^/ NO3^-^** | |
|  | Surgery only | Surgery +Thoracotomy |  |  | Surgery only | Surgery +Thoracotomy |
| **Time** | **n=3** | **n=4** |  | **Time** | **n=3** | **n=4** |
| **Baseline** | 0 ± 0 | 0 ± 0 |  | **Baseline** | 37 ± 9 | 63 ± 21 |
| **0** | 0 ± 0 | 0 ± 0 |  | **0** | 35 ± 13 | 59 ± 19 |
| **30** | 0 ± 0 | 0 ± 0 |  | **30** | 38 ± 13 | 62 ± 19 |
| **60** | 0 ± 0 | 0 ± 0 |  | **60** | 46 ± 17 | 55 ± 17 |
| **90** | 0 ± 0 | 0 ± 0 |  | **90** | 30 ± 9 | 53 ± 19 |
| **120** | 0 ± 0 | 0 ± 0 |  | **120** | 34 ± 16 | 61 ± 23 |
| **150** | 0 ± 0 | 0 ± 0 |  | **150** | 41 ± 12 | 53 ± 15 |
| **180** | 0 ± 0 | 2 ± 0 |  | **180** | 33 ± 12 | 57 ± 21 |
| **210** | 0 ± 0 | 4 ± 4 |  | **210** | 34 ± 12 | 57 ± 21 |
| **240** | 0 ± 0 | 5 ± 5 |  | **240** | 34 ± 11 | 58 ± 23 |
| **270** | 3 ± 2 | 8 ± 8 |  | **270** | 32 ± 12 | 58 ± 28 |
| **300** | 1 ± 1 | 4 ± 4 |  | **300** | 35 ± 12 | 53 ±29 |
| **330** | 5 ± 5 | - |  | **330** | 33 ± 10 | - |
