# Supplementary figures and images for: An Adequately Robust Early TNF-α Response Is a Hallmark of Survival Following Trauma/Hemorrhage
Source: PLoS One. 2009 Dec 22;4(12):e8406. doi: 10.1371/journal.pone.0008406 (PMC2794373; doi:10.1371/journal.pone.0008406)

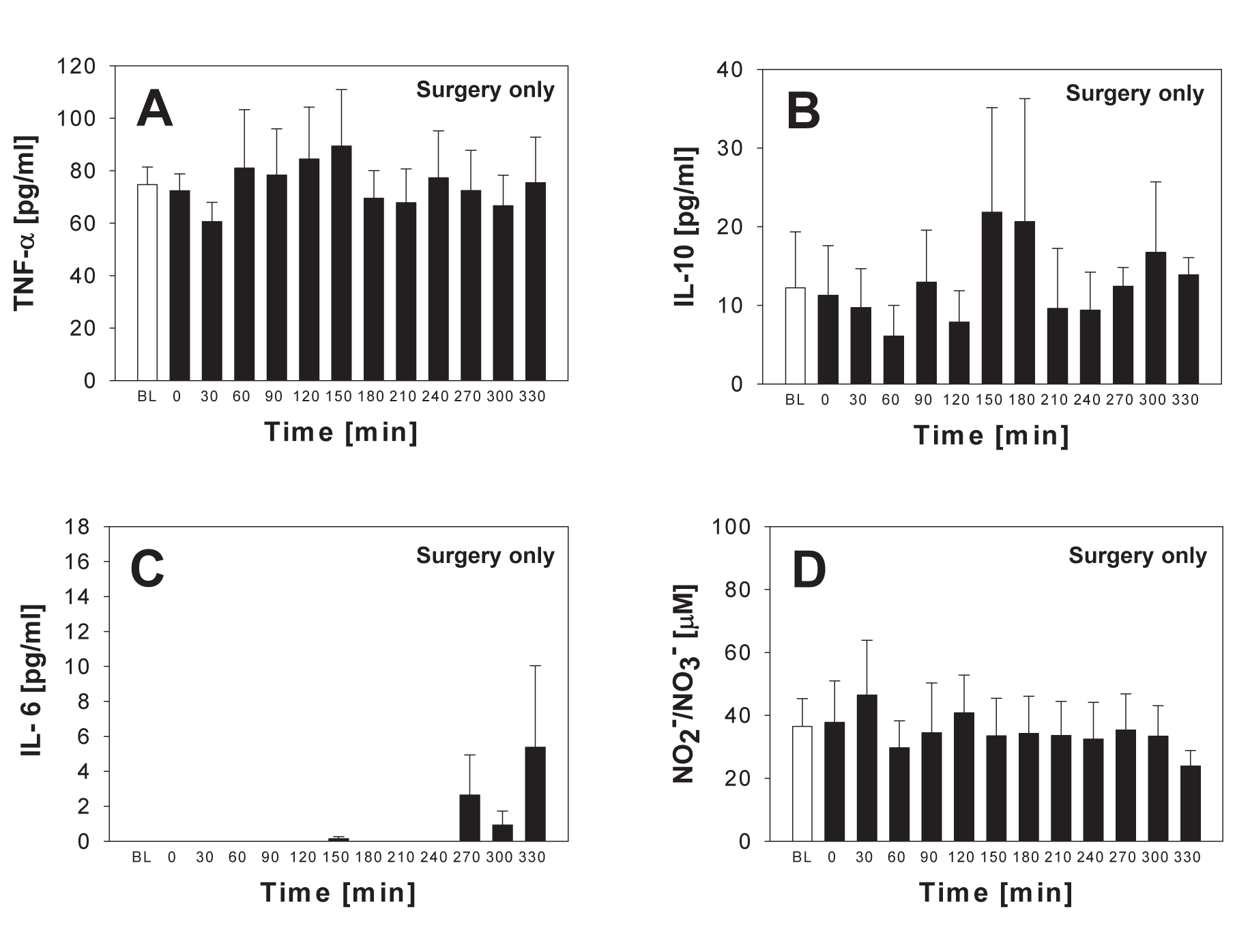

Supplement: Figure S1 — Plasma cytokine and nitrite/nitrate levels in pigs subjected to surgical cannulation only. Plasma samples from 3 swine (all survivors) taken at different time points (see Fig. 2B) were assayed for TNF-α (A), IL-10 (B), IL-6 (C) and NO2 -/NO3 - (D) as described in the Materials and Methods . Results represent the mean±SEM (*P<0.05 vs. baseline, analyzed by One-Way ANOVA followed by the Tukey post hoc test). (0.19 MB TIF) [file pone.0008406.s003.tif]

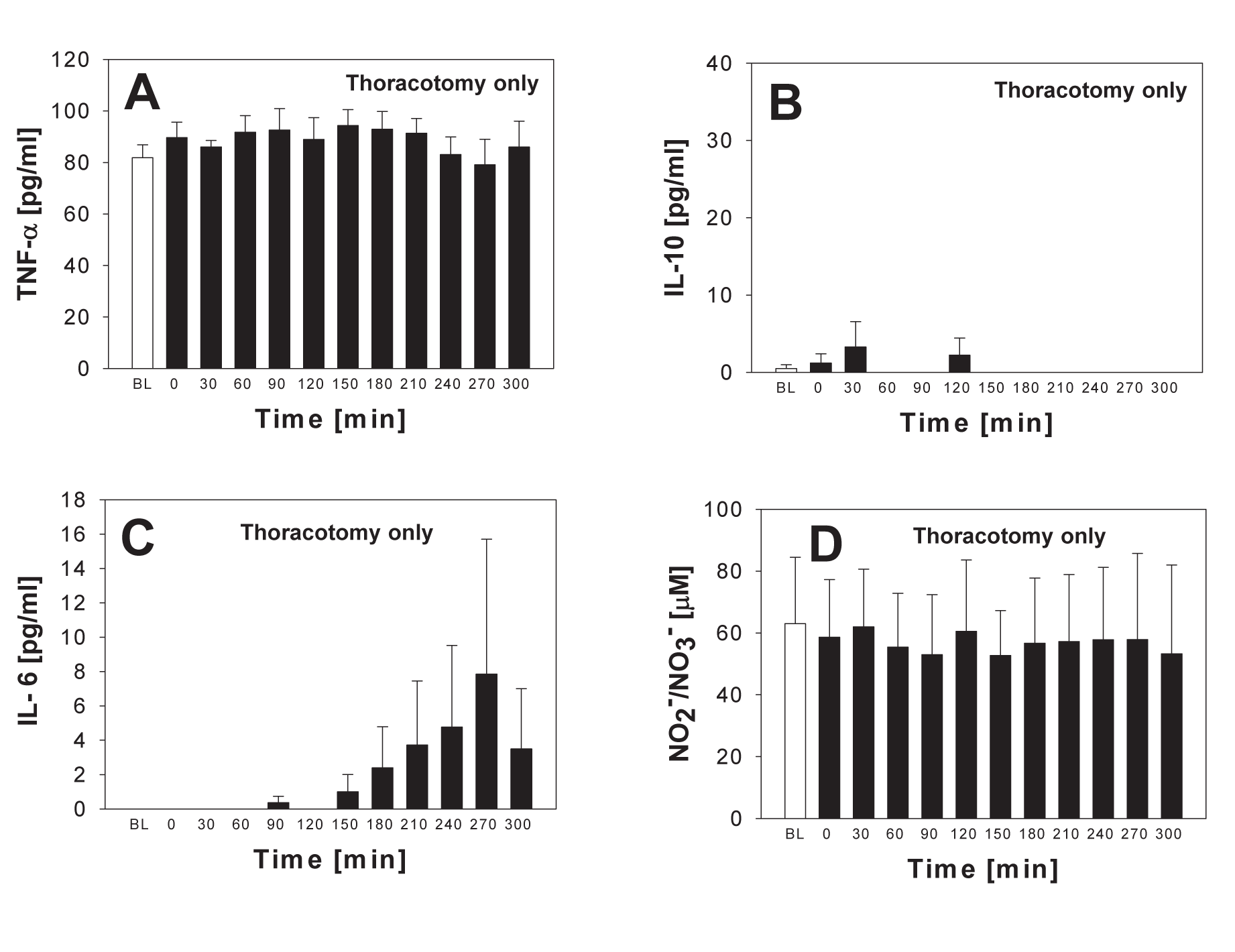

Supplement: Figure S2 — Plasma cytokine and nitrite/nitrate levels in pigs subjected to surgical cannulation in combination with anterolateral thoracotomy only. Plasma samples from 4 swine (all survivors) taken at different time points (see Fig. 2B) were assayed for TNF-α (A), IL-10 (B), IL-6 (C) and NO2 -/NO3 - (D) as described in the Materials and Methods . Results represent the mean±SEM (*P<0.05 vs. baseline, analyzed by One-Way ANOVA followed by the Tukey post hoc test). (0.19 MB TIF) [file pone.0008406.s004.tif]
